# Prefrontal expectancy and reinforcement-driven antidepressant placebo effects

**DOI:** 10.1038/s41398-018-0263-y

**Published:** 2018-10-15

**Authors:** M. Peciña, J. Heffernan, J. Wilson, J. K. Zubieta, A. Y. Dombrovski

**Affiliations:** 1 Department of Psychiatry, University of Pittsburgh, Pittsburgh, PA USA; 2 Department of Neurology, University of Milwaukee, Wisconsin, WI USA; 3 Department of Psychiatry, University of Utah, Salt Lake City, UT USA

## Abstract

Placebo responses in depression exemplify how expectancies and appraisals impact mood. Cognitive and neural mechanisms underlying these responses are still poorly understood, partly due to the difficulty of simulating antidepressant effects and manipulating mood experimentally. To address these challenges, we developed an acute antidepressant placebo experiment involving the intravenous administration of a “fast-acting antidepressant” and a trial-by-trial sham fMRI “neurofeedback” manipulation, purporting to reveal mood-relevant neural responses. Twenty volunteers with major depression underwent this experiment while rating their expected and actual mood improvement. Mixed-effects analyses of trial-by-trial ratings revealed that the “drug” infusion cues induced higher expectancies of mood improvement, while both the “drug” infusion cue and the sham neurofeedback induced a reported mood improvement. Neurofeedback of greater magnitude, compared to lower magnitude, recruited the lateral prefrontal cortex (lPFC). Individuals with greater lPFC responses to neurofeedback displayed: (1) greater effect of previous mood improvement on expectancy ratings and (2) greater effect of sham neurofeedback on mood improvement. Behavioral antidepressant placebo effects were additionally moderated by changes in peripheral β-endorphin plasma levels and depressive symptomatology. These data demonstrate the feasibility of trial-by-trial manipulation of antidepressant placebo-associated expectancies and their reinforcement. We provide initial insights into the role of the lPFC in the interplay between placebo-induced expectancies and mood, as well as preliminary evidence for the role of the opioid system in antidepressant placebo effects.

## Introduction

Mood is shaped by one’s interpretation of reality, including expectancies and appraisals^1^. For example, treatment with drugs that contain no therapeutic ingredient, such as placebos, can alter the subjective perception of mood states. In these cases, the change in perception depends on a prior belief—that the treatment will be effective. However, the precise neural mechanisms through which expectancies modulate mood are currently unknown.

While our understanding of antidepressant placebo effects is limited, much can be learned from the literature on neural mechanisms of placebo analgesia. In many neuroimaging placebo analgesia experiments, participants are given an inert treatment (e.g., a topical cream) along with verbal instructions (“This is a potent analgesic”) that induce expectations for pain relief. This is usually compared to a control condition—the same inert substance without expected pain relief. To reinforce verbal instructions, the placebo is then paired with hidden reduced stimulus intensity during an associative learning, or conditioning, phase. Finally, participants go through a neuroimaging test phase, during which the same stimuli are administered under both the control and the placebo conditions. Experimenters then test whether pain reports and brain responses are modulated by the patient’s beliefs about the treatment^2^. Alternatively, the conditioning phase has been achieved during the pairing of the relevant stimuli (e.g., IAPS pictures, pain stimuli) and an acute active treatment (e.g., analgesic or anxiolytic treatments)^3^. Supporting the notion that expectancies interact with pain perception at a neural level, this line of research has revealed that during placebo analgesia, increased activity in prefrontal regions, including the ventromedial prefrontal cortex (vmPFC), dorsolateral PFC (dlPFC), and lateral orbitofrontal cortex (lOFC), results in reductions in pain-related networks (midcingulate, thalamus, and mid- and anterior insula)^4,5^, modulating endogenous opioid neurotransmission^6–9^, and downstream pain pathways (periaqueductal gray and rostroventral medulla)^10^.

In studies of antidepressant placebo effects, the experimental manipulation of expectations of mood improvement as well as its conditioning posits additional challenges. For example, the delayed action of common antidepressant treatments limits the possibility of acutely manipulating expectancies. Furthermore, mood, unlike pain—which reliably emerges in response to specific stimuli—is a latent state with complex internal dynamics. For these and other reasons, most studies of antidepressant placebo effects have relied solely on verbal instructions in the absence of reinforcement^11–14^. Neuroimaging studies aiming to investigate the neural correlates of antidepressant placebo effects have commonly relied on prospective data from randomized control trials which may be affected by confounds, such as the effects of natural history or regression to the mean. Still, these studies have implicated areas involved in cognitive control (dlPFC) and representations of expectancies and cognitive appraisals (vmPFC, including the rostral anterior cingulate cortex (ACC)^11,13,14^. These studies also supported the role of opioid-mediated neurotransmission in limbic regions^12^.

Here we describe a new paradigm involving trial-by-trial manipulation of two putative components of the placebo antidepressant effect: expectancy of mood improvement (intravenous infusion of a “fast-acting antidepressant”) and conditioning, involving sham neurofeedback, purportedly from a brain area subserving mood. Rapid, trial-by-trial manipulation of expectancies enables us to separate them from intrinsic variability in mood states that is beyond experimental control. Sham neurofeedback, on the other hand, serves as a conditioned reinforcer, akin to the conditioning phase of analgesia studies, by providing a sham read-out of individuals’ brain. Although a primary reinforcer (like pain relief in analgesia) would have been desirable, this is hardly possible during antidepressant treatment, and conditioned reinforcers, such as money or social rewards, reliably modulate mood^15,16^. First, we hypothesized that the intravenous “drug” infusions and sham neurofeedback would induce higher expectancy of improvement and better mood, respectively. Second, we hypothesized that, as in the case of placebo analgesia, antidepressant placebo effects would involve modulation of areas implicated in the representation of expectancies, cognitive appraisal (vmPFC), cognitive control (dlPFC) and stimulus–outcome mapping (ventrolateral prefrontal cortex (vlPFC), lOFC). Furthermore, we hypothesized that activation in these regions will predict placebo-induced mood improvement. Third, following-up on our previous work regarding the role of the central opioid system in antidepressant placebo effects^12^, we reasoned that placebo-induced changes in opioid neurotransmission could be detected peripherally, indexed by an increase in β-endorphin plasma levels. Accordingly, we hypothesized that peripheral increases in β-endorphin plasma levels will scale with blood-oxygen-level dependent (BOLD) responses to the experimental neurofeedback manipulation. Finally, we investigated how placebo responses were moderated by depressive symptoms.

## Methods

### Participants

Twenty-four patients diagnosed with major depressive disorder (MDD) were recruited through the UMClinicalStudies.org website and enrolled after signing informed consent (aged 18–50 years: mean = 27.67 ± 8.37; 19 females). All patients were un-medicated, right-handed, fluent in English, and had a current primary diagnosis of non-psychotic MDD per the Mini-International Neuropsychiatric Interview (M.I.N.I)^17^ with or without anxiety disorders. Exclusion criteria included*:* pregnancy or breastfeeding; history of psychotic depressive, schizophrenic, bipolar, schizoaffective, or other psychotic disorders; meeting M.I.N.I. criteria for substance dependence in the last 6 months, except for nicotine, or substance abuse in the last 2 months; requiring immediate hospitalization for a psychiatric disorder or an unstable general medical condition; actively suicidal or considered a high suicide risk, or having any contraindication for the performance of the magnetic resonance imaging (MRI). After enrollment, three participants were excluded (two were claustrophobic and one could not complete the study due to scanner maintenance issues). Another participant was excluded due to an incidental finding, and therefore data were available in 20 patients (mean age = 28.8 ± 8.6; 16 females).

### Authorized deception and manipulation of the patient’s expectations

Patients were informed that the purpose of the study was to investigate the effects of a fast-acting intravenous antidepressant treatment on real-time brain signal in order to understand the neural mechanisms implicated in fast-acting antidepressant responses. In order to allow the use of deception, the consent form included the following statement: *“You should be aware that the investigators have intentionally mis-described certain aspects of the study that will be revealed to you at the end of participation. This use of deception is necessary to obtain valid results. However, an independent research committee has determined that this consent form describes all major risks and benefits of the study. The investigator will explain the mis-described aspects of the study to you at the end of your participation”*^25^.

#### Clinical assessments

Depressive symptoms were assessed using a structured interview, the Montgomery–Åsberg Rating Scale (MADRS) and the 17-item Hamilton Depression Rating Scale (HDRS-17)^18,19^. Because of our interest in reward and motivation, core symptoms of depression, heavily involved in placebo responses^20,21^, we investigated different facets of reward-guided behavior^22^ using two different questionnaires: the Apathy Evaluation Scale (AES) (motivation)^23^ and the Snaith–Hamilton Pleasure Scale (SHAPS) (reward sensitivity)^24^.

### β-Endorphin assay

Blood samples were collected in 17 subjects at two time-points: 1 h before the scanner and immediately at the end of the scanner (data were missing in three subjects). β-Endorphin plasma levels were extracted using the human β-Endorphin assay (Elabscience, E-EL-H0572) which employs the quantitative sandwich enzyme immunoassay technique. The assay had a range (standard curve) of 15.63–1000 pg/mL. We report the following coefficients of variation (CVs): inter-assay CV: 12.8% at 85 pg/mL and 10.2% at 252 pg/mL; intra-assay CV: 5.1% at 85 pg/mL and 6.2% at 252 pg/mL.

### Design overview section

#### Sham neurofeedback fMRI task

**Fig. 1:**
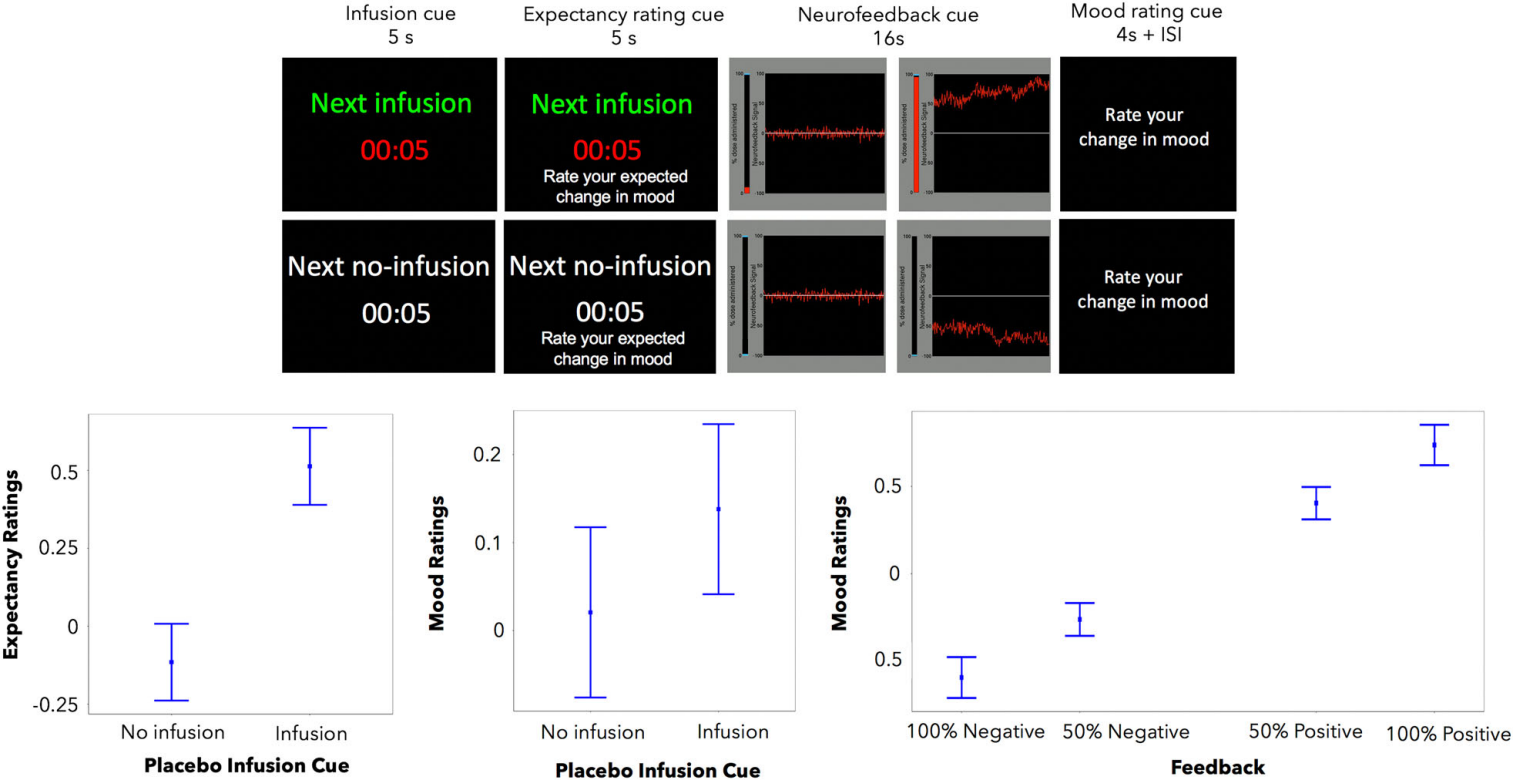
**Sham neurofeedback task.** Top: During each trial, participants are presented with either a “drug” infusion or no-infusion cue, followed by a positive or negative neurofeedback cue of two different magnitudes of response, low and high. Bottom: The “drug” infusions, compared to the no-infusions, were associated with significantly higher expectancy and mood ratings (left, center). Similarly, positive sham neurofeedback, compared to negative, was associated with significantly higher mood ratings (right). *Y-*axis indicates expectancy or mood ratings (much better = 3, better = 2, slightly better = 1, no change = 0, slightly worse = −1, worse = −2, and much worse = −3). Error bars represent 95% confidence intervals

The task featured a within-subject trial-by-trial (interleaved) manipulation of two putative components of the placebo antidepressant effect: the expectancy of mood improvement and its reinforcement (Fig. 1). Expectancy was manipulated using a drug infusion or no-infusion cue, which instructed patients of an imminent infusion of the “fast-acting antidepressant” or the absence of drug delivery, respectively. Reinforcement was manipulated through the presentation of sham neurofeedback, which reflected sham increased (positive tracing) or decreased (negative tracing) brain activity in response to each drug infusion and no-infusion cues, respectively.

Before the scanning session, patients watched a fragment of a movie that would be displayed again during the sham neurofeedback functional MRI (fMRI) task. This movie instructed participants to expect multiple “drug” infusions of the “fast-acting antidepressant” (the intravenous saline placebo), followed by the display of the recorded brain signal (sham neurofeedback). Patients were instructed that each drug administration would start immediately after each infusion cue, but that a delay of a few seconds should be expected before changes in brain signal could be detected. Subjects were told that positive neurofeedback signal (higher tracing) indicated the effectiveness of the “drug” infusions and could be associated with subsequent mood improvements. In addition, patients were taught to expect no-infusion periods, as the experimental control condition. No-infusion periods were followed by negative neurofeedback (tracing going below 0) and were described as normal physiological responses in the absence of the drug infusions that could lead to mood worsening. Positive and negative neurofeedback signal were displayed at two different magnitudes, low and high, and participants were instructed that the greater the magnitude of the signal the higher the effect of the drug infusion.

After each infusion period, participants were asked to rate their expected and actual change in mood on a 7-point Likert scale with the following anchors and coding: much better = 3, better = 2, slightly better = 1, no change = 0, slightly worse = −1, worse = −2, and much worse = −3.

An fMRI intravenous compatible line was placed in the participant’s arm immediately before entering the scanner. An MRI compatible pump, controlled from outside the scanning room by pushing the “go” trigger, delivered the saline to the participant during the scanning session. The infusion was manually started at a given flow rate and volume, at the beginning of each run.

Once in the scanner, the Sham neurofeedback fMRI task consisted of six 7-min scanner runs of 12 trials each.

Inter-trial intervals were jittered. Jitter duration was randomly sampled from a discrete distribution of 2, 3, or 4 s. No jitter length could be selected more than 4 times per run. Infusion and feedback were presented in a pseudorandom order which was the same for all participants.

Placebo-associated expectancies were manipulated using the infusions of “fast-acting antidepressant”. During the infusion trials, participants were presented with a “next infusion” cue (10 s). During the no-infusion trials, participants were presented with a similar “no infusion” cue.

Conditioned reinforcement was intended to experimentally induce trial-by-trial variation in mood and consisted of sham neurofeedback, with a 2 × 2 valence (positive and negative) and magnitude (high, low) manipulation.

To prevent placebo-associated expectancies from being quickly extinguished, we presented positive feedback on 67% of infusion trials and 33% of no-infusion trials and vice versa. We ascertained that the effects of these factors on mood were statistically dissociable, since correlation between the fixed effects of infusion cue and the neurofeedback was only modest (*r* = 0.298).

Subjects’ expectancies and mood improvement ratings were assessed on every trial by responding to the questions: “Rate your expected change in mood”, and “Rate your change in mood”. Participants used a keypad and their index fingers to respond using the “Patient’s Expectations/Impression of Improvement Questionnaire” as described above.

#### Assessment of the credibility of the experiment

After the experiment, the investigators assessed the task’s credibility by asking the following questions: *From 0 to 100% how often: (1) did the neurofeedback signal reflect your brain activity, (2) was the fast-acting antidepressant treatment given to you during the infusion periods? and (3) was saline given to you during the no-infusion periods?* Subjects who responded 0 to questions 1 and 2 and responded 100 to question 3 were excluded from the experiment (*n* = 0).

#### Image acquisition and data processing

Functional MR images were acquired at the University of Michigan Hospital on a 3T scanner (Philips Achieva, Best, The Netherlands) using a single-shot echo-planar imaging (EPI) sequence (repetition time (TR) = 2000, echo time (TE) = 35 ms, flip angle (FA) = 90, field of view (FOV) = 20 cm, 64 × 64 matrix, voxel size = 8 mm^3^). The task, implemented in PsychToolbox-3^26^, was presented to subjects via a display placed behind the gantry. In each of the 6 runs of the task, 228 volumes were acquired. Two sets of *anatomical MR images* were acquired on the same scanner, including a high-resolution MPRAGE scan for co-registration to the Montreal Neurological Institute (MNI) template and a functional-resolution scan to improve standardization. Functional images were preprocessed using FSL (version 5.0.2.2, http://www.fmrib.ox.ac.uk), SPM8 (http://www.fil.ion.ucl.ac.uk), and MATLAB software (version 7.14.0.739). Images were slice-time corrected (middle reference slice) and realigned to the mean of the time series data. All datasets were reviewed for motion and sessions were excluded if head motion between consecutive frames was greater than 3 mm in any direction. The high-resolution T1-weighted image was co-registered to the functional images using a boundary-based registration algorithm (FSL FLIRT), segmented into tissue probability maps (FSL FAST), and normalized to MNI space (FSL FNIRT). Functional images were normalized with FSL FNIRT deformation parameters, resampled to isotropic 2-mm voxels, and smoothed with a 6 mm Gaussian kernel (SPM8).

#### fMRI: statistical analysis

Image analyses were performed using AFNI (17.0.01). For subject-level analysis, regressors included infusion/no-infusion onsets, expectancy rating onsets, the inflection of neurofeedback signal onsets, and the mood improvement rating onsets. All events were modeled at “onset”. Infusion and feedback event regressors were modeled using hemodynamic response function (HRF)-convolved boxcar functions of 6 and 8 s duration, respectively, whereas expectancy and mood rating regressors were reaction time (RT) convolved. Additionally, first-order parametric regressors were constructed in order to model the effect of expectancy (“drug”-infusion vs no-infusion), valence (positive versus negative neurofeedback), and magnitude (high versus low tracing), where each was weighted 1 and −1, respectively. The null model was a fourth-order polynomial. Voxelwise BOLD signal was regressed on these estimates in the single-subject analyses using AFNI 3dDeconvolve. All runs for each subject were concatenated during first-level modeling with the 3dDeconvolve auto-concatenate default. Group analyses for each regressor employed AFNI 3dttest++. To avoid assumptions about the special autocorrelation function, the cluster threshold for whole-brain analysis was set non-parametrically (3dttest++ with the –clustsim option), yielding *k* = 376 at the voxelwise *p* *<* 0.005. Data from statistically significant clusters from whole-brain level analyses were extracted and used as regressors in the linear mixed-effects (LME) analysis. We used the Talairach–Tournoux atlas for region identification.

### Behavior: statistical analysis

Effects of experimental manipulations and subject characteristics on expectancy and mood ratings were estimated in hierarchical LME regression using the lme4 package in R^27^. Models were fit using Restricted Maximum Likelihood (REML). Fixed effects for the prediction of expectancy ratings included: infusion/no-infusion cues, previous mood ratings, and their two-way interactions. Subject and run (clustering within-subject) intercepts were taken to be random in all models. Fixed effects for the prediction of mood ratings model included: infusion/no-infusion cues, expectancy ratings, reinforcement cues (high positive = 2; low positive = 1; low negative = −1, high negative = −2) and their two-way interactions. We tested for significance of predictors using the likelihood ratio test (LRT; car:Anova^28^), after refitting the model using full ML (instead of REML). To diagnose multicollinearity among predictors we calculated variance inflation factors (VIFs) for predictors, as suggested by Zuur et al.^29^. We ascertained that all regressors met a rigorous criterion, VIF < 2.

An additional set of LMEs evaluated main effects of regional BOLD activation (measured by mean regression coefficients from significant clusters), β-endorphin measures, and depression severity scores on expectancy and mood ratings, and most importantly their interactions with experimental manipulations. Moderation effects on each of these separate models were determined by the statistical significance of the interaction between BOLD activation, depression severity scores, β-endorphin measures, and the task-related predictors within the LME models. Model comparisons relied on LRT (for nested models) and Akaike information criterion (AIC). The model including β-endorphin measures was not compared against the others due to the reduced number of subjects in this model.

## Results

### Placebo-induced expectancies

Patients’ expectancy ratings were significantly higher during the placebo infusion condition (expecting a drug infusion as opposed to no infusion). Similarly, better mood ratings in the previous trial were significantly associated with higher expectancy ratings (Table 1, model 1a; Fig. 1). The two-way interaction between these predictors was not significant.

**Table 1:** Expectancy and feedback ratings mixed-effects models

	Dependent variable: expectancy ratings					
	Model 1a		Model 2a		Model 3a	
**Fixed effects**	b	S.E.	b	S.E.	b	S.E.
(Intercept)	−0.11	0.05	−0.17	0.07	−0.1	0.05
Placebo cue	0.63***	0.04	0.63***	0.04	0.63***	0.04
Previous mood rating	0.01*	0.02	−0.01	0.03	0.02*	0.02
Placebo cue × previous mood rating	0.06	0.03	0.06	0.03	0.05	0.03
vlPFC/dlPFC			0.01	0.05		
vlPFC × placebo cue			0.06	0.04		
vlPFC × previous mood rating			0.05*	0.02		
Anhedonia					0.17*	0.04
Anhedonia × placebo cue					−0.15***	0.04
Anhedonia × previous mood rating					−0.01	0.02
Observations	1218		1218		1218	
Akaike Inf. Crit.	2583.2		2578		2566	
Df.	1221		1209		1209	
	**Dependent variable: mood ratings**					
	**Model 1b**		**Model 2b**		**Model 3b**	
**Fixed effects**	b	S.E.	b	S.E.	b	S.E.
(Intercept)	−0.03	0.04	−0.02	0.04	−0.02	0.04
Placebo cue	0.12*	0.06	0.11*	0.05	0.11*	0.06
Expectancy ratings	0.22***	0.04	0.23***	0.04	0.22***	0.04
Feedback cue	0.32***	0.02	0.32***	0.02	0.32***	0.02
Feedback cue × expectancy ratings	0.07***	0.02	0.06***	0.02	0.06***	0.02
vlPFC/dlPFC			0.05	0.03		
vlPFC/dlPFC × expectancy ratings			−0.07*	0.03		
vlPFC/dlPFC × feedback cue			0.2***	0.01		
Anhedonia					−0.04*	0.04
Anhedonia × placebo cue					0.001	0.06
Anhedonia × expectancy ratings					−0.07*	0.03
Anhedonia × feedback cue					0.05***	0.02
Df.	1228		1226		1225	
Observations	1236		1236		1236	
Akaike Inf. Crit.	3232.6		3103.5		3222.5	

*b* estimate, *S.E*. standard error, *Df*. degrees of freedom of the residual, *vlPFC/dlPFC* ventrolateral/dorsolateral prefrontal cortex

*p < 0.05; **p < 0.01; ***p < 0.001

### Placebo effect on mood

As expected, patients reported greater mood improvement during the infusion cue, compared to the no-infusion cue, and following the display of positive sham neurofeedback, compared to negative. Higher expectancies also predicted higher mood ratings. The positive effect of neurofeedback on reported mood was enhanced when expectancies were high, as reflected in a positive two-way interaction (Table 1, model 1b; Fig. 1).

### Neural responses to the anticipation of drug infusion and during the infusion and sham neurofeedback

**Fig. 2:**
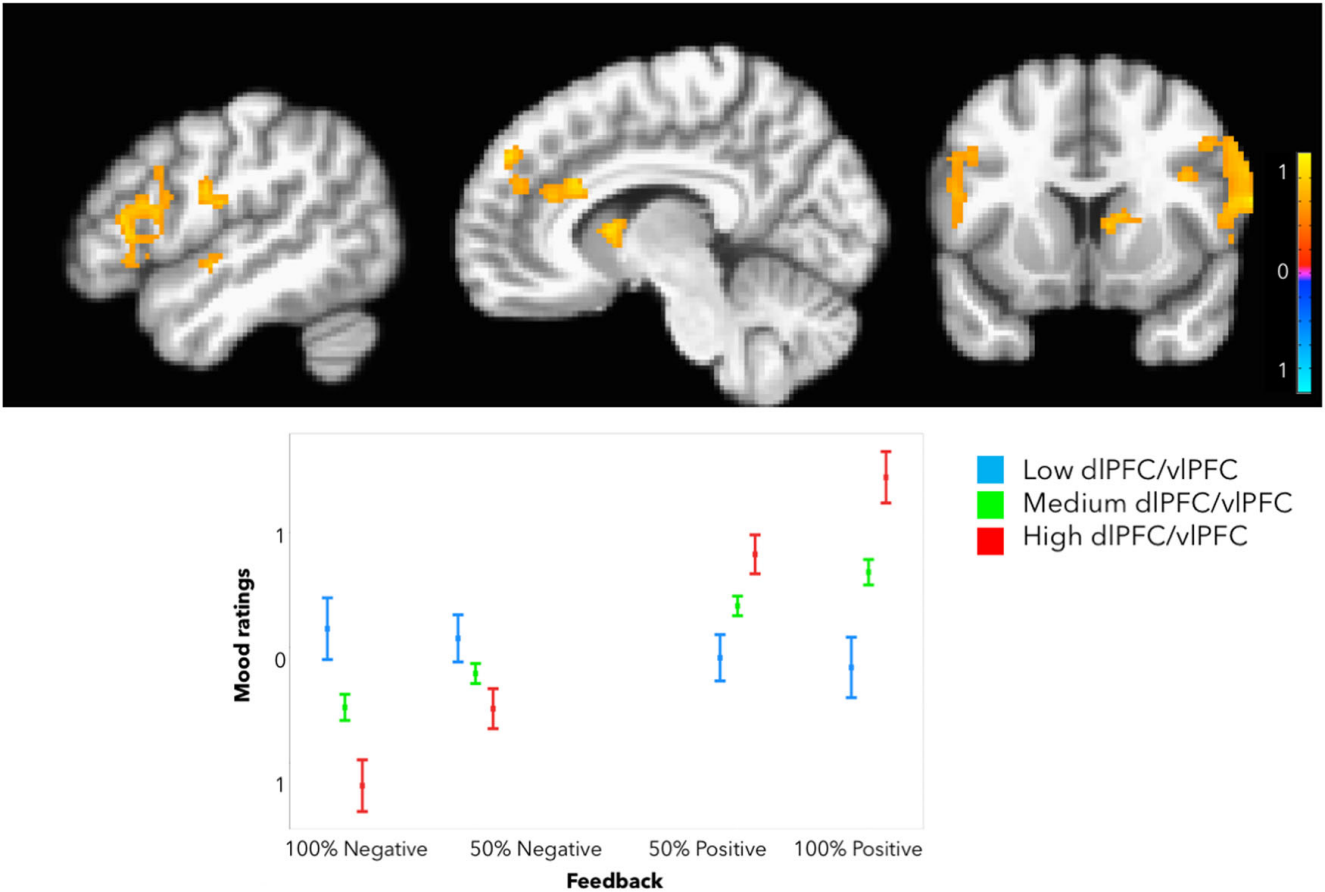
**Placebo-induced neural responses in the vlPFC.** Top: Neurofeedback of greater magnitude, regardless of valence, was associated with significantly greater BOLD responses in the vlPFC/dlPFC. Bottom: Greater vlPFC/dlPFC responses were associated with lower mood improvement. *Y*-axis indicates expectancy or mood ratings (much better = 3, better = 2, slightly better = 1, no change = 0, slightly worse = −1, worse = −2, and much worse = −3). Error bars represent 95% confidence intervals

We found a significant effect of neurofeedback magnitude. The presentation of positive neurofeedback of greater magnitude (high>low) recruited greater BOLD responses in the bilateral vlPFC/dlPFC. Smaller clusters in the rostral/dorsal ACC and the ventral striatum did not survive correction for multiple comparisons. vlPFC/dlPFC extracted parameter estimates were taken into subsequent LME analyses (Fig. 2).

We found no significant modulation by the infusion cue, compared to the no-infusion cue, or the neurofeedback valence (positive vs. negative).

### Brain–behavior relationships

Increased BOLD responses in the left vlPFC/dlPFC during sham neurofeedback were associated with greater expectancy ratings when better mood ratings were reported in the previous trial, as reflected by the positive left vlPFC/dlPFC response × previous mood rating interaction (Table 1, model 2a; Fig. 2).

A right vlPFC/dlPFC × Infusion cue interaction (estimate = −0.06, S.E. = 0.03, *t* = −1.8, *p* = 0.06) and right vlPFC/dlPFC × previous mood rating interaction (estimate = −0.06, S.E. = 0.02, *t* = 1.8, *p* = 0.06) fell short of statistical significance.

Higher BOLD responses in the bilateral vlPFC/dlPFC during sham neurofeedback were associated with better mood ratings following “positive neurofeedback”, as reflected by a positive vlPFC/dlPFC response by neurofeedback interaction. However, they negatively moderated the effect of higher expectancies on subsequent mood improvement, as reflected by a negative vlPFC/dlPFC by expectancy ratings interaction (Table 1, model 2b; Fig. 2).

### Peripheral opioid responses and mood

**Fig. 3:**
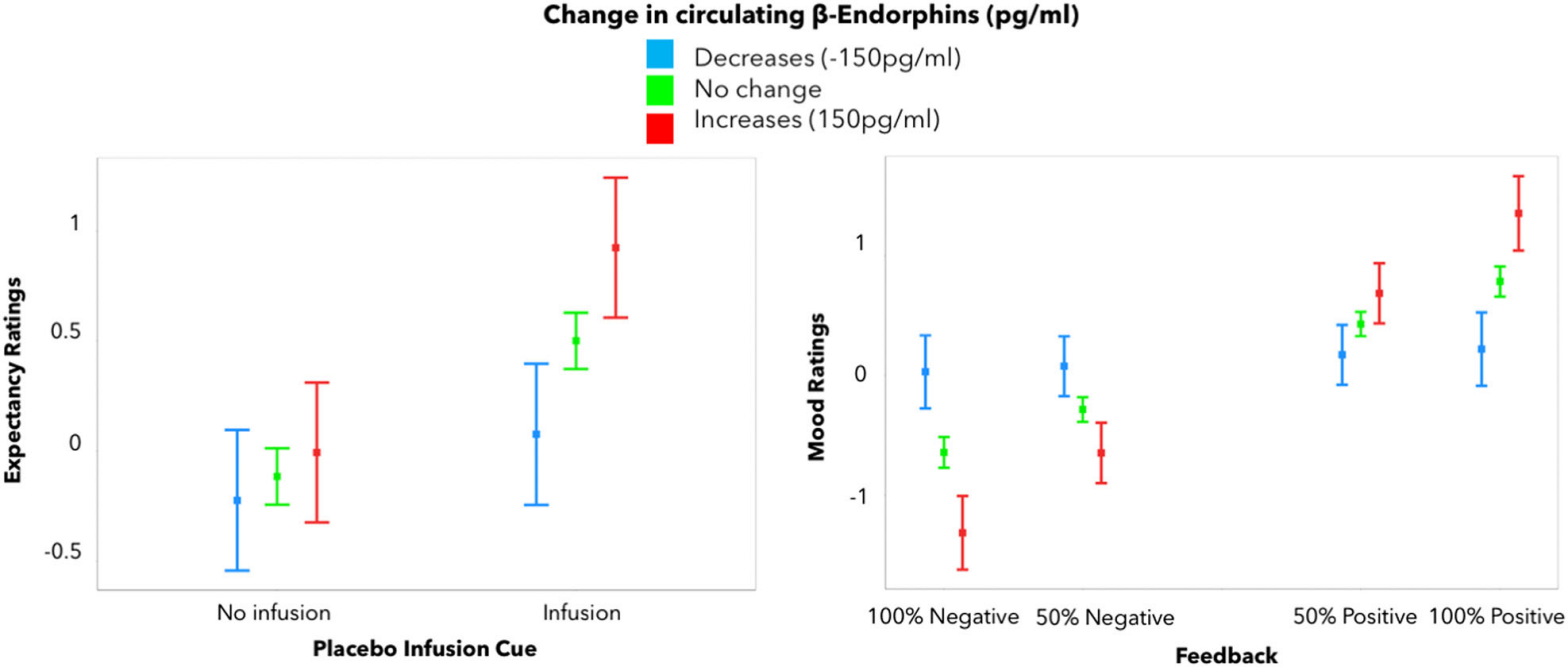
**Placebo-induced changes in β-endorphins.** Greater increases in β-endorphin plasma levels following the experiment were associated with higher expectancy of improvement on placebo (left) and mood ratings (right). *Y*-axis indicates expectancy or mood ratings (much better = 3, better = 2, slightly better = 1, no change = 0, slightly worse = −1, worse = −2, and much worse = −3). Error bars represent 95% confidence intervals

We then investigated whether our placebo manipulation was associated with significant changes in β-endorphin plasma levels. In the entire group, β-endorphin plasma levels did not increase significantly following the experiment (pre = 32.8 ± 10.4 pg/mL; post = 38 ± 8.6 pg/mL). However, greater increases in β-endorphin plasma levels following the placebo experiment were associated with significantly greater expectancy ratings (estimate = 0.0007, S.E. = 0.0008, *t*_df=18 _= 0.9, *p* = 0.02), as well as a significant placebo infusion cue  by changes in β-endorphins interaction (estimate = 0.002, S.E. = 0.0006, *t*_df=18 _= 3.2, *p* = 0.001), and greater subjective mood improvement in response positive neurofeedback (estimate = 0.002, S.E. = 0.002, *t* = 7.8, *p* < 0.001) (Fig. 3). Contrary to our hypothesis, changes in β-endorphins were not significantly correlated with vlPFC neural responses to neurofeedback.

### Impact of depressive symptoms

We then examined whether depressive symptoms, in particular, anhedonia and apathy, predicted changes in expectancy and mood ratings during the task. HDRS-17, Snaith–Hamilton Anhedonia, and AES scores were highly correlated and therefore added separately into the LME models. Models that included the Snaith–Hamilton Anhedonia scores are reported here, and those that included the HDRS-17 and the AES scores are reported in Supplemental Table 1.

**Fig. 4:**
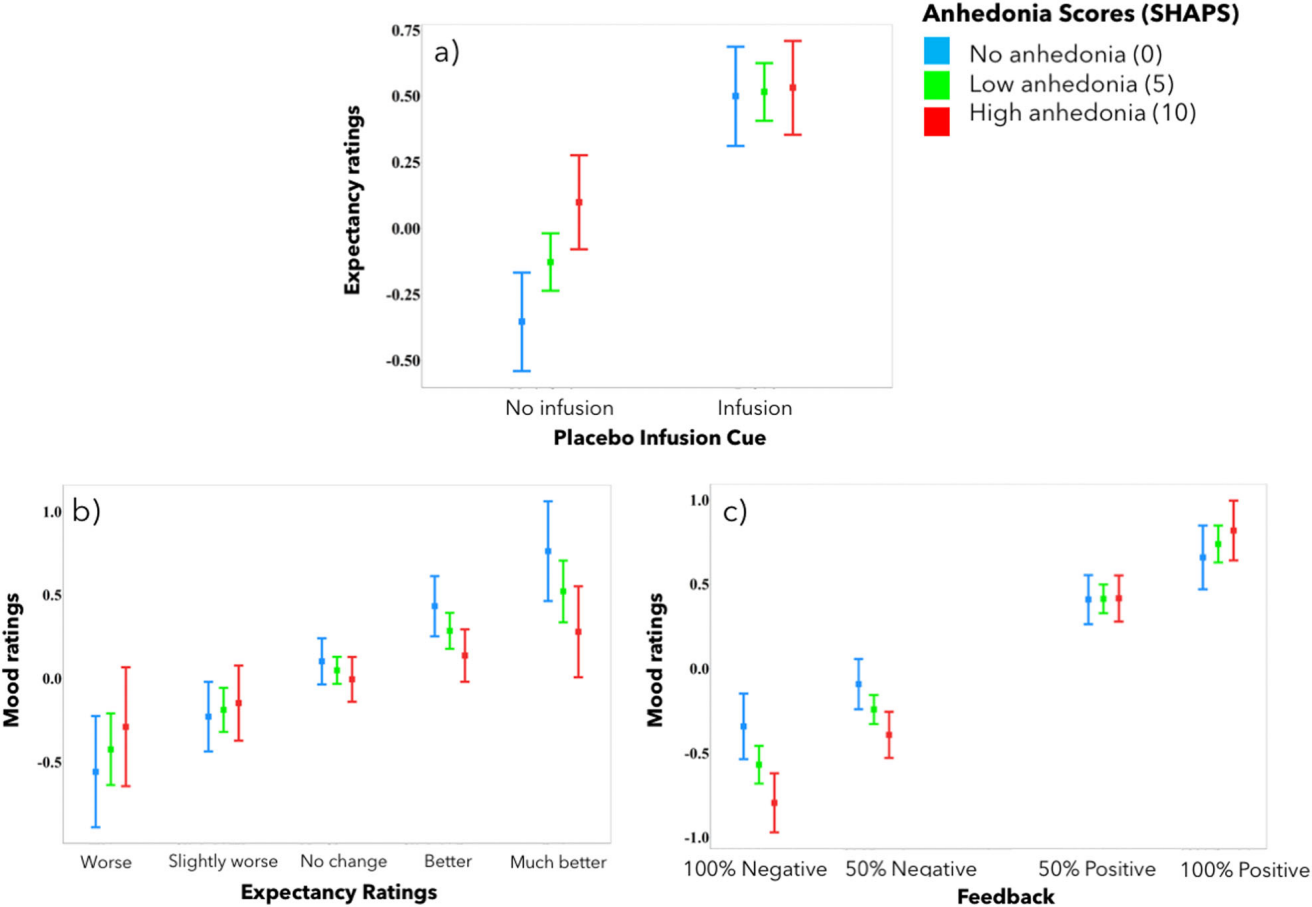
**Anhedonia and expectancy and mood ratings.** Patients with higher self-reported anhedonia scores expressed greater expectations of improvement in response to the no-infusion cue (**a**). High anhedonia scores also predicted lower impact of expectancies on mood ratings (**b**) but greater mood modulation by neurofeedback (**c**). *Y*-axis indicates expectancy or mood ratings (much better = 3, better = 2, slightly better = 1, no change = 0, slightly worse = −1, worse = −2, and much worse = −3). Error bars represent 95% confidence intervals

High anhedonia predicted weaker modulation of expectancy ratings by infusions, which was mostly due to higher expectancy ratings in the control condition in anhedonic participants (Table 1, model 3a, Fig. 4a).

High anhedonia scores also predicted weaker modulation of mood by expectancies (Table 1, model 3b, Fig. 4b) and stronger modulation by neurofeedback (Table 1, model 3b, Fig. 4c). Thus overall, anhedonia predicted diminished infusion cue-related effects and accentuated effects of placebo experience (neurofeedback).

## Discussion

In this study, we examined the role of expectancies and their reinforcement in depressed patients receiving a placebo described as a fast-acting antidepressant during the display of sham neurofeedback with expectations of drug effectiveness. Confirming the general hypothesis that antidepressant placebo effects depend on expectancies and their reinforcement, as suggested from the field of placebo analgesia^30–33^ and antidepressant efficacy clinical trials^34–36^, our experimental manipulation demonstrated that the drug infusions in patients who reported mood improvement in the previous trial were associated with significantly higher expectancy ratings. The placebo infusion and the sham neurofeedback cues, as well as higher expectancy ratings, were associated with higher self-reported mood improvement.

Our expectancy and reinforcement manipulation of antidepressant placebo effects resulted in increased BOLD responses in the lateral PFC (vlPFC and dlPFC). This pattern of activation was specific to the display of neurofeedback of greater magnitude, compared to the one of lower magnitude, regardless of the valence of the neurofeedback signal. The vlPFC plays a key role in the encoding of stimulus–outcome mapping during decision making and attentional processes^37,38^. Accordingly, the vlPFC receives highly processed visual information from the inferior temporal cortex, as well as inputs from the amygdala, OFC, and other outcome-responsive structures^39,40^. With one exception^41^, this region has not been implicated in placebo analgesia, although it seems to play a key role in the reduction of negative effect during cognitive appraisal of aversive images^42^, which supports its more specific role in antidepressant placebo effects. The dlPFC, on the other hand, has been strongly associated with the emergence of placebo analgesia^5,43,44^ as well as placebo effects in social pain^41^, especially during the anticipation of the placebo experience^5^ and the subjective expectancies^44^. Furthermore, it has been suggested that the mechanisms of action of transcranial direct-current stimulation, a common treatment for depression, might at least partly be explained by the stimulation of dlPFC-mediated expectancy networks^45^. A recent study has also linked placebo-induced neural activity in the dlPFC to the improvement of affective ratings in subjects exposed to a recent romantic rejection^41^. In our study, vlPFC/dlPFC activation was associated with stronger coupling between preceding mood improvement and current expectancy. Increased modulation of the bilateral vlPFC/dlPFC was also associated with a diminished effect of expectancy ratings on current mood, potentially highlighting a tradeoff between the effects of moment-to-moment experiences and prior expectancies on mood.

Somewhat surprisingly, we found no significant effects of neurofeedback valence. This is consistent with the notion that reinforcers share common neural pathways, regardless of valence^46,47^. In fact, vlPFC activation has been observed in response to both positive and negative emotion-induction tasks—often in tandem with subcortical activation^48^—and its activity increases in response to both decreasing and increasing negative emotion via reappraisal^49,50^. Reappraisal theory has indeed been invoked to explain the formation of placebo analgesia^51^.

Placebo-associated expectancies were coupled with peripheral changes in β-endorphin levels, as indicated by the effect of increased peripheral β-endorphin release on expectancy ratings, especially in response to the “drug” infusion cue. At the same time, greater increases in β-endorphin levels were also paralleled by increased mood in response to the sham neurofeedback experience, suggesting a generally heightened sensitivity to reward-predicting stimuli. This finding parallels a number of studies demonstrating the involvement of the central opioid system in placebo analgesia^4,9,52–54^ and more recently in the development of antidepressant placebo effect^12^. One previous study found increases in plasma β-endorphins during an ischemic pain challenge (tourniquet), but found no significant differences between the placebo, nocebo, and natural history groups^55^. These increases in β-endorphin plasma levels were interpreted in the context of a stress-induced analgesia response. Still, we found no significant relationship between the changes in circulating β-endorphins and vlPFC/dlPFC responses to the experience of neurofeedback, which suggests that peripheral β-endorphin levels do not directly relate to synaptic endogenous opioid system function in the PFC, and might influence placebo responses through alternative mechanisms.

While our experiment did not include a group of non-depressed controls, which precluded us from conclusively isolating the aspects of placebo effect unique to depression, the effects of depressive symptoms in general, and anhedonia in particular, on expectancies and mood improvement highlight the aspects of the placebo effect potentially unique to depression. First, higher anhedonia scores resulted in greater mood changes in response to sham neurofeedback and smaller effects of the prior expectancy on mood. These effects are consistent with the idea that in patients with depression, anhedonia is primarily characterized by reductions in appetitive learning rather than the primary sensitivity to rewards, as suggested by a recent computational model-based study of reinforcement learning in patients with depression^56^. Alternatively, it may be argued that in our experiment, patients with high anhedonia scores had more at stake, making the mood improvement even more desirable in the more severely affected patients. This interpretation is supported by the fact that negative neurofeedback had particularly strong effects in patients with high anhedonia scores, potentially indicating disappointment. Second, high anhedonia predicted lower modulation of expectancies by the placebo infusion cue, although this effect was primarily driven by the responses to the anticipation of the no-infusion cue and might again be explained by motivational effects in more severe cases.

This study is a direct demonstration of the cognitive and neural mechanisms involved in expectancy- and reinforcement-induced antidepressant placebo effects. Our study yields preliminary support to the hypotheses that: 1) expectancies of fast-acting antidepressant effects and their reinforcement with sham neurofeedback alter mood by modulating the lateral prefrontal cortex, and 2) that the endogenous opioid system subserves certain aspects of these responses. These preliminary findings raise interesting questions for future research: Are positive expectancies and their reinforcement simply additive to subsequent mood experiences, or do they transform the way those experiences are encoded? Specifically, do such expectancies modulate new learning from reinforcement or introduce an appraisal bias? The answers to these questions will inform our interpretation of data from studies of fast-acting antidepressants. Furthermore, experimental manipulations of expectancies and reinforcement will enable us to leverage Bayesian reinforcement learning models for interrogating neural systems involved in placebo response.

## Electronic supplementary material

Supplemental Material
