# Supplementary material for: Prefrontal expectancy and reinforcement-driven antidepressant placebo effects
Source: Transl Psychiatry. 2018 Oct 15;8:222. doi: 10.1038/s41398-018-0263-y (PMC6189213; doi:10.1038/s41398-018-0263-y)
Supplement: Supplementary file 1 — Supplemental Material [file 41398_2018_263_MOESM1_ESM.docx]

**Prefrontal expectancy and reinforcement-driven antidepressant placebo effects.**

**Supplemental Material**

^1^Peciña M., ^2^Heffernan J., ^1^Wilson J., ^3^Zubieta J.K., ^1^Dombrovski A.Y.

^1^Department of Psychiatry, University of Pittsburgh, Pittsburgh, PA, USA.

^2^Department of Neurology, Univesity of Milwaukee, Wisconsin, WI, USA.

^3^Department of Psychiatry, University of Utah, Salt Lake City, UT, USA.

**Supplemental Table 1: Mixed-effects model of clinical measures on expectancy and mood ratings**
